# Expression of aberrant antigens in hematological malignancies: A single center experience

**DOI:** 10.12669/pjms.342.13996

**Published:** 2018

**Authors:** Aneeta Shahni, Madiha Saud, Saima Siddiqui, Samina Naz Mukry

**Affiliations:** 1 Aneeta Shahni, BS (Clinical Laboratory Sciences). National Institute of Blood Diseases & Bone Marrow Transplantation, ST 2/A Block 17 Gulshan-e-Iqbal KDA Scheme 24, Karachi, Pakistan; 2 Madiha Saud, M.Sc. National Institute of Blood Diseases & Bone Marrow Transplantation, ST 2/A Block 17 Gulshan-e-Iqbal KDA Scheme 24, Karachi, Pakistan; 3 Saima Siddiqui, MBBS, FCPS. National Institute of Blood Diseases & Bone Marrow Transplantation, ST 2/A Block 17 Gulshan-e-Iqbal KDA Scheme 24, Karachi, Pakistan; 4 Samina Naz Mukry, Ph.D. National Institute of Blood Diseases & Bone Marrow Transplantation, ST 2/A Block 17 Gulshan-e-Iqbal KDA Scheme 24, Karachi, Pakistan

**Keywords:** Aberrant CD markers, Monoclonal antibodies (MoAbs), Multi-parametric Flow-Cytometry

## Abstract

**Background and Objective:**

Aberrant phenotype is a phenomenon of abnormal expression or loss of expression of cell specific lineage marker not associated with specific cell type. Aberrant phenotype expression due to genetic defects may be associated with unfavorable outcome. It can be used to determine minimal residual disease status. The purpose of the study was to find out the occurrence of aberrant phenotypes in leukemia/lymphoma patients.

**Methods:**

One milliliter peripheral blood or bone marrow samples were analyzed on FACS Calibur flowcytometer. The cells were lysed and stained following standard protocol. Data was acquired and analyzed by CellQuest-Pro software. The Antigenic expression was rated as positive when the percentage of positive blast cells was ≥ 20%. In that manner, aberrant phenotype was considered positive when 20% of blast cells show expression of markers.

**Results:**

Of a total 145 cases analyzed, 26 were acute myeloid leukemia, 71 of acute lymphoblastic leukaemia, 48 were of Chronic Lymphoid leukemia on the basis of morphological features and confirmed by flow cytometry. Overall, 19% (28) cases showed aberrant expression of antigens. In 32% (9/28) AML patients, CD5, CD7, CD64dim, CD10, CD117, CD25 and TdT were expressed while in 25% (7/28) ALL patients CD33, CD13, HLA-DR and CD3 were detected. Among chronic leukemia, all aberrant expressions were seen in cases of B-CLL (10/28) only; with CD11c, CD3 and CD10 as the aberrantly expressed markers.

**Conclusion:**

Variability in aberrant phenotype expression was observed in different types of acute and chronic leukemia patients with no prognostic implications on treatment response.

## INTRODUCTION

The cancer due to defective differentiation of heamatopoietic stem cells is categorized as hematological malignancies. In these malignancies uncontrolled abnormal cells proliferation occur due to genetic aberration which repress normal hemopoiesis resulting in altered physiological activities.^1^,^2^ These defects might be of myleoid or lymphoid lineage.^3^

Leukemia is diagnosed by morphology and cytochemical examination of blast cells, immunophenotyping, cytogenetic and molecular genetics further help in confirmation. Since, leukemia in general is regarded as a defect of cell differentiation; therefore, the flow cytometry/immunophenotyping identifies cell surface/ CD markers, expressed at different stages of cell development, by applying monoclonal antibodies against them helps in the diagnosis and classification of leukemia. The morphologically similar blast cells can be easily differentiated by immunophenotyping on the basis of expression and different CD (cluster of differentiation) markers. The flowcytometric analysis can be completed within hours in virtually any case and is often sufficient.^4^-^6^

Commonly expressed CD markers on T-lymphocytes include CD2, CD3, CD5, CD7 while CD19, CD20, CD22, CD79a are common markers expressed on B cell. Similarly, CD 13, CD33 and MPO are markers of myeloid lineage of cells. Each marker has its own lineage specific role. For instance, CD3 is the lineage-specific marker for T cell lympho-proliferations; physiologically it helps in signal transduction.^7^ The pan T-cell antigens i-e CD5 and CD7 are markers specific to both mature and immature T-cells and help in immune regulation.^7^ CD 10 or CALLA is a zinc dependent metalloprotease and is helpful in diagnosis of childhood ALL on cycling cells with propensity to apoptosis. CD11c or α_x_ integrin is another marker expressed on neutrophils monocytes, and some subsets of lymphocyte it mediate cell-cell and cell-matrix interaction.^8^ The clinically significant markers CD13 and CD33 are early markers of myeloid lineage expressed in very initial stage of differentiation even before expression of any morphologic sign of myeloid differentiation.^9^ If any of these markers express their presence in other lineage rather than their own they are considered as aberrant markers. More precisely, aberrant phenotype in general is a phenomenon of abnormal expression or loss of expression of specific cell lineage markers not associated with particular cell type like for example the expression of lymphoid CD markers on myeloblasts and vice versa.^10^ Abnormal gene defects results in the expression of aberrant immunophenotypes. Aberrant expression also helps in sub-classifying the type of leukemia.

The detection of aberrancies in newly originating blood cells by flow cytometry is of diagnostic and prognostic importance. Aberrant expression of antigens may be associated with adverse outcomes.^11^ Minimal residual disease (MRD) in leukemic patients may also be detected on the basis of these antigens. Detection of aberrant markers is of extreme importance in the decision of a tailored treatment regimen and thus the eventual outcome of the patients. The present study was conducted to determine the frequency of aberrant phenotypes in leukemia/lymphoma patients.

### Patient Selection

A total of 145 patients were included in this study from June (2013) to December (2016). By following International Council for Harmonization (ICH) guidelines for Good Clinical Practice [ICH-GCP] informed consent was obtained from all patients. After morphological diagnosis of leukemia, immunophenotyping was done for confirmation using either blood or bone marrow sample. The cytogenetic analysis was performed from heparinized bone marrow aspirates for AML and ALL cases.

### Treatment protocol

AML patients received 3+7 protocols induction with (Daunorubicin for three days and cytarabine for seven days) followed by consolidation with either high dose Cytosar or FLAG-IDA and some patients were on supportive care. Patients diagnosed with T-cell ALL received induction with either Hyper CVAD protocols or poor risk UK ALL XI. B-cell ALL received UK ALL followed by early and late intensification or FLAG-IDA. Patients with B-cell CLL received FC-R or FC protocol and supportive care.

### Immunophenotyping by FACS

The cells were lysed and stained following standard protocol. Briefly, 1ml of whole blood/bone marrow was added to 10ml of 1X BD FACS lysing solution in 15 ml. falcon tubes. The mixture was gently mixed and incubated at room temperature (22-25°C) for 30 minutes. Tubes were centrifuged at 500g for 5 min. About 10 ml PBS was used to wash the cell pellet thrice. Cells were then resuspended in adequate volume of PBS to achieve a concentration of 1 million cells/10µl, approximately, one million cells were carefully transferred to BD falcon tubes and 10µl of monoclonal antibodies conjugated with FITC, PE, PerCP or APC were added to appropriately marked tubes. The tubes were then incubated in the dark for 30-60 minutes. Cells were washed again to remove excess antibodies with PBS as described earlier. Finally, about 300 µl of 1% paraformaldehyde solution was added to fix cells. The stained cells were stored at 2-8°C until acquisition by FACS Calibur (Becton, Dickinson Company, CA, USA). For intra-cytoplasmic markers cells were pre-treated with permeabilizing solution for 10 minutes, after washing same procedure was followed as for surface markers. IgG1 isotype controls for each flourochrome were also included in the assay. Data was acquired by CellQuest Pro software while analysis was done by Paint-a-gate software. The antigenic expression was rated as positive when the percentage of positive blast cells was ≥ 20% either in aberrant or conventional case. Similarly, aberrant phenotypes were defined when at least 20% of the blast cells expressed that particular phenotype. A separate defined panel of monoclonal antibodies was used for acute and chronic leukemia (Table-I).

**Table-I:** Panels of antibodies for immunophenotyping of acute and chronic leukemia.

Acute leukemia	Chronic leukemia
CD3	CD3
CD4	CD4
CD5	CD5
CD7	CD7
CD8	CD10
CD10	CD19
CD13	CD20
CD19	CD23
CD20	CD25
CD33	CD45
CD34	CD79a
CD45	ZAP70
CD64	KAPPA
CD79a	LAMBDA
CD117	FMC7
MPO	
TDT	

### Karyotyping by cytogenetics

Cytogenetic analysis for karyotyping was performed in AML and ALL cases on overnight, 24-hr unstimulated BM cultures and 72-hr stimulated BM cultures using standard procedures. The slides were stained using G-bands via trypsin using Giemsa banding technique. The International System for Human Cytogenetic Nomenclature (ISHCN) 2013was followed. In each case 15 metaphases were analyzed to make karyogram using Cytovision Sys.

### Statistical analysis

The SPSS version 17 was used for statistical analysis. The descriptive parameters were used in terms of mean±standard deviation. The Chi square test (χ^2^) was applied to determine the possible significance of occurrence of aberrant phenotype in our study population; values <0.05 were considered as significant.

## RESULTS

A total of 97 cases diagnosed as acute leukemia (26 of AML & 71 of ALL) and 48 patients as chronic leukemia (36 of Leukemia and 12 of lymphoma) were tested by flow cytometry. The median age of patients was 32(1.5-82yrs). AML classification was done on the basis of FAB nomenclature. Patient’s characteristics are summarized in Table-II. Twenty eight (19%) cases showed aberrant expression of CD antigens (Table-III). Out of 28, 19 patients with aberrant phenotype were male with a female: male ratio of 1:1.5. The cytogenetic abnormalities with aberrant CD markers expression was observed in only two patients suffering from acute leukemia. The unusual expression of CD7 and CD 64 with cytogenetic abnormality 46, XX, del(9q21:23.3) was observed in AML (M2)while in an ALL patient aberrant expression of CD10, CD13 and CD117 with cytogenetic abnormality 46, XX/46, XY was detected.

**Table-II:** Patients characteristics.

Characteristics	Mean±SD
Age	46.5±20
Hemoglobin	9.3±2.6
RBC Count	5.3±26.5
WBC Count	87.1±39.2
Platelets	102±79.3

**Table-III:** An overview of aberrant and conventional phenotype in patients of hematological malignancies.

Disease	No. of cases	Aberrant cases	Conventional cases
AML	26	9	17
ALL	71	7	64
CLL	46	10	36
B-cell Non-Hodgkin lymphoma	2	2	0

### Aberrant phenotypes in AML patients

**Fig.1:**
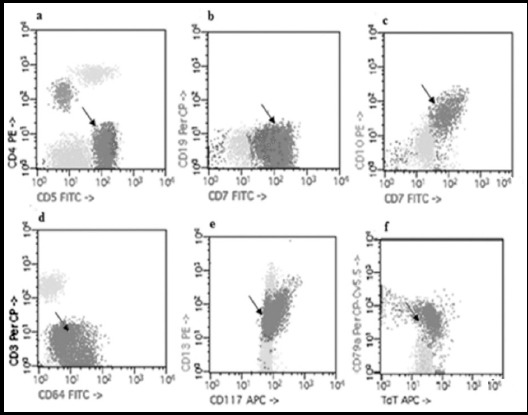
Aberrant expression of markers in AML; arrow shows aberrantly expressed (a): CD5 marker, (b):CD7 marker, (c): CD10 marker, (d):CD64 marker, (e): CD117 marker and (f) TdT marker.

About 34% (9/26) of AML patients exhibited aberrant phenotype i.e. expression of lymphoid markers during our study. In Acute Myeloid Leukemia with minimal differentiation CD7, CD10 were aberrantly expressed. In Acute Myeloid Leukemia without Maturation CD5, CD7, TdT were aberrantly expressed. In Acute Myeloid Leukemia with maturation CD64 was expressed which was uncommon. In Acute Myelomonocytice Leukemia only CD 7 was expressed. CD117 was aberrantly expressed in Acute Monoblastic Leukemia. Most commonly expressed markers included CD5, CD7, CD64dim, CD10, CD117 and TdT with *p* value <0.05(Fig.1). The AML cases were further sub-classified following FAB nomenclature. Details of aberrant phenotypes in properly sub-classified cases are summarized in Table-IV.

**Table-IV:** Expression of Aberrant markers in FAB classified AML.

FAB	CD5	CD7	CD10	CD64	CD117	TdT
M_0_	-	2	1	-	-	-
M_1_	1	1	-	-	-	4
M_2_	-	-	-	1	-	-
M_3_	-	-	-	1	-	-
M_4_	-	1	-	1	-	-
M_5_	-	-	-	-	1	-
M_6_	-	-	-	-	-	-
M_7_	-	-	-	-	-	-

### Aberrant phenotype in ALL patients

**Fig.2:**
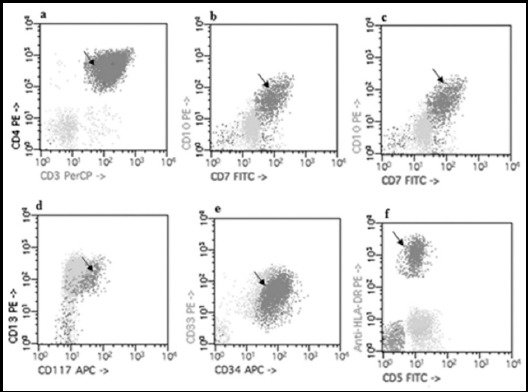
Aberrant expression of in ALL; arrow shows aberrantly expressed (a) CD3 marker (b) CD7 marker (c) CD10 marker (d) CD13 marker (e) CD33 marker (f) Anti-HLA-DR marker.

**Fig.3:**
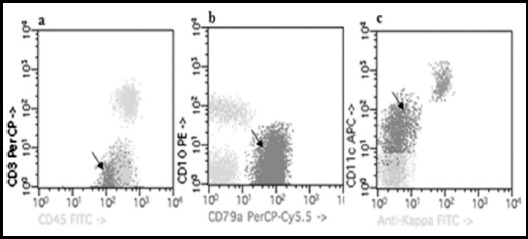
Aberrant expression of B-cell CLL; arrow shows aberrantly expressed (a) CD3 marker (b) CD10 marker (c) CD11c marker.

Only seven out of 71 ALL patients expressed aberrant phenotype. Approximately, 10% patients with aberrant phenotype expressed CD3, CD7, and HLA-DR with *p* value <0.05 during the present study. Most frequent aberrant marker in ALL was CD13 followed by CD33 as shown in Fig.2. It is also noteworthy that only markers of myeloid origin were expressed as aberrant phenotype in T-cell ALL (CD13, CD33 and HLA-DR) while common T-cell markers i.e. CD3 and CD7 were aberrantly expressed in 3% cases of B-cell ALL/ TAg+ B-ALL. About 2% cases of B-cell ALL co-expressed markers of T-cell (CD7) and myeloid origin (CD13 and CD33) both (Fig.3). In one case of Relapsed ALL there was expression of CD3 as an aberrant marker.

### Aberrant phenotype in CLL patients

Overall 21% (10/48) of CLL cases showed aberrant expression of CD3, CD10 and CD11c (Fig.4). The CD3 and CD10 (T-cell markers) were observed in 2% cases (1/48) of B-cell CLL only (p= <0.05). The most commonly expressed marker was CD11c with a frequency of 17% (p= <0.05).

### Aberrant phenotype in B-cell non-Hodgkin lymphoma patients

Abnormal expression of CD8 marker (p= <0.05) was observed in the 2 cases of B-cell non-Hodgkin lymphoma included in the present study.

### Aberrant phenotype and drug response

Based on our observation no association was found between the response to treatment and the expression of aberrant phenotype. About 83.87% cases responded well to the treatment while only 16.13% were non-responders. The mortality rate was 40% (2/5) among the non-responders with aberrant phenotype.

## DISCUSSION

Leukemia is a condition of abnormal cell differentiation resulting in high number of blast cells with abnormal cell function.^12^ During the course of normal hematopoiesis the precursor cells originate from stem cells expressing developmental stage specific cell surface markers. These cell surface markers are generally taken as phenotypic markers to detect the exact stage of cell differentiation defect to classify hematological conditions like leukemia.^3^ The aberrantly expressed markers are important in management of complicated cases of leukemia.^13^

The lymphoid marker CD7 is documented as the most frequently expressed aberrant marker in AML by Zhang et al 20% and Jehadi et al. 31%. Similarly, the most common markers observed during the present study were CD7 and TdT as their expression was observed in 44.4% (4/9) patients with aberrant phenotype. It was interesting to note that CD7 were found in 40% while TdT in 44% patients of AML with minimal differentiation (M1). A comparatively low frequency (22%) of TdT was reported by Mazhar et al.^14^ in AML. CD117 was only observed in a single case of M5 while no aberrancy in phenotypes was observed in any cases of M6 and M7. One case of acute myelomonocytic leukemia (M4) showed expression of CD7 and CD64 along with cytogenetic abnormality del9 (q21-23.3). The aberrant phenotype expression was less frequent in AML cases i.e. 34% compared to previously reported literature where even 88% aberrant expression was reported in patients suffering from AML.^15^,^16^

The frequency of aberrant expression in ALL was 10% (7/71) which was lower than the results of Paietta et al.^17^ i.e. 21.2%and 39% claimed by Bhushan et al.^18^, Hussain et al., 2011 also studied the expression of T-ALL specific markers in B-ALL and 15% cases (4/26) of T-cell ALL showed CD13, CD33 and HLA-DR expression in their study.^19^

A few reviews have been published regarding CD10 appearance in B-cell CLL, however the actual frequency about CD10 expression in cases on CLL/SLL is not known.^20^,^21^ Kampalath et al studied the prognostic significance of CD10 in CLL/SLL and reported a comparatively higher frequency of 10.3%.^22^ The CD11c is a marker of acute myelomonocytic leukemia; it was the most common aberrant marker observed in about 17% B-CLL cases (8/48). This observation is in line with previous studies where 22-27% cases of B-CLL exhibited strong CD 11c expression.^18^,^19^

Unfortunately, in Pakistan there is no registered data for the incidence of non-Hodgkin lymphoma (NHL). But in a study Shahid et al mentioned that NHL is the four times more common cancer in the males as compare to females which is 6.1% in Pakistan.^23^ In our study, there were only two cases of non-Hodgkin lymphoma and both showed aberrant expression of CD8 marker which is in line with study by Carulli et al.^24^ The significance of CD8 as prognostic marker is still under debate with contradictory studies on its association with aggressive form of disease.

Despite the aberrant expression of markers the response to treatment was good in 83.87% cases while only 16.13% i.e. 5 patients were non-responders. Among these 40% (2/5) were females while 60% (3/5) were male. Similar findings were reported by Jahedi et al where aberrant phenotype had no prognostic implications. Few studies also suggest that aberrant expression is associated with the poor outcome of leukemia.^25^ During this study such poor outcome was also observed in two out of five non-responders and both were females who expired during the course of treatment.

## CONCLUSION

Based on our study, it can be concluded that heterogeneous immunophenotypes are less common i.e. 28% in our specified group of hematological malignancies. Furthermore, if present these phenotypes, which are actually a result of abnormal genetic programme, can be used as a marker for management of such patients. It can also be suggested that these uniquely expressed markers cannot be taken as a determinant of aggressive form of disease with little or no response to treatment protocols as observed. Further studies with larger numbers would help in validating and clarifying the prognostic role of such aberrant expression profiles.
